# CD47 blockade inhibits tumor progression human osteosarcoma in xenograft models

**DOI:** 10.18632/oncotarget.4282

**Published:** 2015-06-08

**Authors:** Ji-Feng Xu, Xiao-Hong Pan, Shui-Jun Zhang, Chen Zhao, Bin-Song Qiu, Hai-Feng Gu, Jian-Fei Hong, Li Cao, Yu Chen, Bing Xia, Qin Bi, Ya-Ping Wang

**Affiliations:** ^1^ Department of Orthopedics and Joint Surgery, Zhejiang Provincial People's Hospital, Hangzhou 310014, PR China; ^2^ Department of Cardiology, Second Affiliated Hospital, College of Medicine, Zhejiang University, Hangzhou 310009, PR China

**Keywords:** osteosarcoma, CD47, antibody, immunotherapy

## Abstract

Osteosarcoma is the most common bone tumors in children and adolescents. Despite intensive chemotherapy, patients with advanced disease still have a poor prognosis, illustrating the need for alternative therapies. In this study, we explored the use of antibodies that block CD47 with a tumor growth suppressive effect on osteosarcoma. We first found that up-regulation of CD47 mRNA levels in the tumorous tissues from eight patients with osteosarcoma when compared with that in adjacent non-tumorous tissues. Further western-blot (WB) and immunohistochemistry (IHC) demonstrated that CD47 protein level was highly expressed in osteosarcoma compared to normal osteoblastic cells and adjacent non-tumorous tissues. Osteosarcoma cancer stem cell markers staining shown that the majority of CD44^+^ cells expressed CD47 albeit with different percentages (ranging from 80% to 99%). Furthermore, high CD47 mRNA expression levels were associated with a decreased probability of progression-free and overall survival. In addition, blockade of CD47 by specific Abs suppresses the invasive ability of osteosarcoma tumor cells and further inhibits spontaneous pulmonary metastasis of KRIB osteosarcoma cells *in vivo*. Finally, CD47 blockade increases macrophage phagocytosis of osteosarcoma tumor cells. In conclusion, our findings demonstrate that CD47 is a critical regulator in the metastasis of osteosarcoma and suggest that targeted inhibition of this antigen by anti-CD47 may be a novel immunotherapeutic approach in the management of this tumor.

## INTRODUCTION

Osteosarcoma is the most common histological form of primary bone cancer, which shows most prevalent in children and young adults [1]. Although about 90% of patients are able to have limb-salvage surgery, 30–40% of patients experience disease relapse and manifest pulmonary metastasis [2]. In addition, only 20% of patients can survive three years after relapse [3]. Based on this clinical status, there is a clear and urgent need to explore and develop new therapies aimed at preventing osteosarcoma development and its pulmonary metastasis.

Immunomodulation to promote anti-tumor effects has been used in clinical use. By targeting surface antigens expressed on tumor cells, specific antibodies have demonstrated efficacy as cancer therapeutics. Recent successful antibody-based strategies (PD-1 and CTLA-4) provided solid evidence on enhancing antitumor immune responses by targeting immune cells, irrespective of tumor antigens [4]. In addition, activation of macrophage anti-tumor activity has been also an area of active investigation, in particular regarding the blockade of the binding between CD47 molecules and macrophage signal regulatory protein-alpha (SIRPα). CD47 is a transmembrane protein that acts as a self signal on normal cells by inhibiting macrophage phagocytosis when CD47 binds to macrophage SIRPα [5]. Higher expression of CD47 was also found in various malignancies, which was considered as a mechanism by which cancer cells increase their “selfness”, thus evading macrophage tumoral activity [6–8]. Furthermore, patients with osteosarcoma tumors that had higher CD47 expression fared poorly [9, 10]. These associations suggest the potential utility of CD47 blockade in the treatment of osteosarcoma. To address this deficiency, we sought to evaluate the therapeutic effects of CD47 blockade using specific antibodies against osteosarcoma progression. We found that CD47 blockade could inhibit tumor growth in the xenograft models of osteosarcoma. Furthermore, CD47 blockade results in a significant increase in tumor phagocytosis by macrophages.

## RESULTS

### CD47 is highly expressed in osteosarcoma

**Figure 1:**
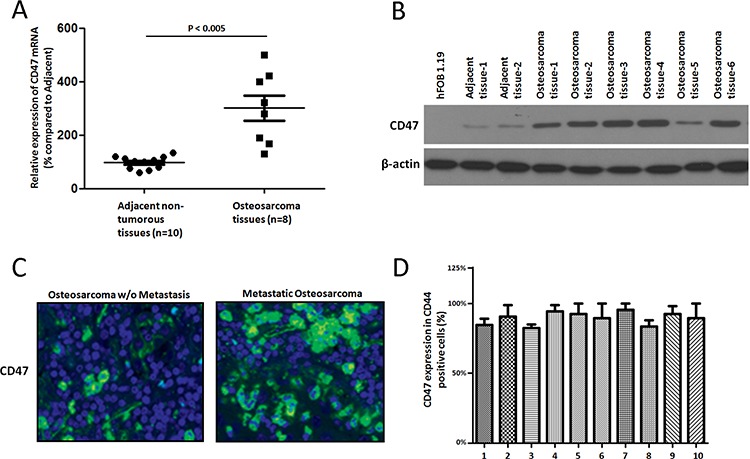
CD47 is highly expressed in osteosarcoma Quantitative RT-PCR **A.** and immunoblot analysis **B.** showed CD47 expression in ten freshly isolated osteosarcoma tissues and their adjacent nontumorous tissues. Beta-actin was treated as the reference control. CD47 immunostaining **C.** showed higher of CD47 expression in osteosarcoma of the same samples with or without metastasis and their adjacent nontumorous tissues. The tissues were counterstained with DAPI Fluorescent Stain. Representative images are shown here. (magnification × 100). **D.** Flow-cytometry analysis of CD47 and CD44 expressions on osteosarcoma tumor cells and quantification of CD44 cells also expressing CD47.

To get a first insight into the role of CD47 in osteosarcoma, we evaluated CD47 expression in freshly isolated osteosarcoma tissues and adjacent non-tumorous tissues. We found that up-regulation of CD47 mRNA levels (Figure 1A) and protein (Figure 1B) levels in the osteosarcoma tissues when compared with those of normal osteoblastic cell lines and adjacent non-tumorous tissues. However, there was no difference in beta-actin expression at the mRNA and protein levels. To find how CD47 expression was regulated during different stages of osteosarcoma, we characterized CD47 reactivity in 40 paraffin-embedded osteosarcoma samples with different degrees of invasive behavior (20 osteosarcoma samples). We could observe strongly up-regulation of CD47 in 17 out of 20 osteosarcoma patients (Figure 1C) compared to those without invasive features (*n* = 10) (*p* < 0.001, Fisher's test). There were no significant difference of other clinic-pathological characteristics like gender and age (data not shown). Because there were no other DNA mutations and no dysregulated methylation variation found in the promoter of CD47 gene by direct sequencing and bisulfate PCR-sequencing, we concluded that up-regulation of CD47 likely occurs at the transcriptional level and CD47 up-regulation was associated with osteosarcoma metastasis. We next assessed the percentage of CD47^+^ cells within the CD44, [a well-established osteosarcoma cancer stem cell (CSC) markers [11], subpopulation in a set of ten primary patient-derived osteosarcoma cancer cell cultures, and as shown in Figure 1D, the majority of CD44^+^ cells expressed CD47 albeit with different percentages (ranging from 80% to 99%), which indicated that which indicated that osteosarcoma CSCs are mostly confined to CD47^+^ cells. These data suggested that targeting CD47 may achieve a reduction on the activity in osteosarcoma cancer stem cells.

### CD47 mRNA expression levels predict survival

**Figure 2:**
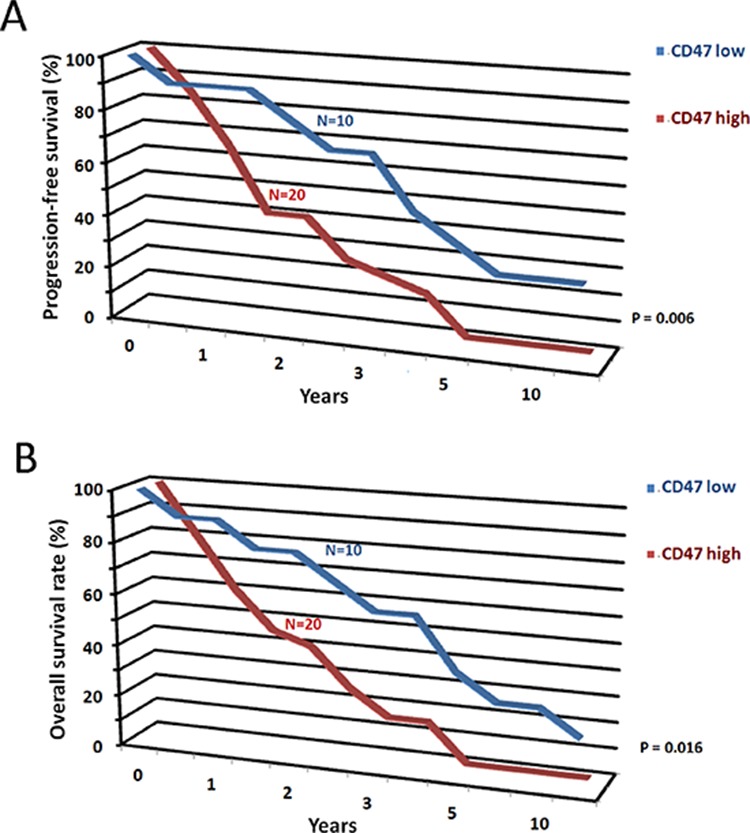
CD47 mRNA expression levels predict survival CD47 mRNA expression levels may be a prognostic factor in osteosarcoma. Increased levels of CD47 mRNA expression were correlated with decreased probability of progression-free survival of osteosarcoma **A.** and overall survival of osteosarcoma **B.**

To determine if CD47 mRNA expression levels were a prognostic factor in patients with osteosarcoma, we analyzed gene-expression data from a cohort of 30 patients with osteosarcoma. In a univariate analysis, stratification of patients into “CD47 high” (*n* = 20) and “CD47 low” (*n* = 10) groups based on an optimum threshold revealed that high CD47 mRNA expression levels were associated with a decreased probability of progression-free (Figure 2A) and overall (Figure 2B) survival. These results suggest that CD47 expression levels may be a clinically relevant prognostic factor in osteosarcoma.

### Effect of anti-CD47 Abs on osteosarcoma cell invasion *in vitro*

**Figure 3:**
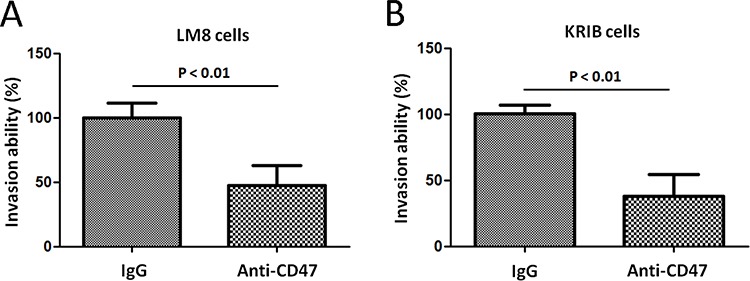
Effect of anti-CD47 Abs on osteosarcoma cell invasion *in vitro* Migration of LM8 cells **A.** and KRIB cells **B.** in spheroid migration assays. Data are given as mean ± s.e.m. Experiments in (A and B) were repeated at least three times.

We next analyzed whether anti-CD47 Abs could inhibit the invasion of osteosarcoma cells through Matrigel-coated filters. Murine osteosarcoma cell line, LM8 cells and human osteosarcoma cell line, KRIB cells that had been treated with either anti-CD47 Abs (B6H12, 100 μg/ml) or control IgG antibody for 4 days were placed in the upper compartment of an invasion chamber in the presence of either anti-CD47 Abs (100 μg/ml) or IgG antibody. After 22 h, the cells on the lower surface were counted. Osteosarcoma cells treated with anti-CD47 Abs exhibited significantly less invasion than did IgG-treated cells (LM8: *P* < 0.001, Figure 3A and KRIB: *P* < 0.001, Figure 3B). These results indicated that blockade of CD47 by specific Abs inhibits the invasive ability of osteosarcoma tumor cells during tumor intravasation and extravasation. We further used the MTT cell proliferation assay to measure cell viability after incubation with IgG control, B6H12 and Ab400 antibodies. Within each antibody concentration and duration of exposure, there was no significantly difference of the viability of normal osteoblastic cells and osteosarcoma tumor cells treated with CD47 blocking antibody (B6H12 and Ab400), IgG and no antibody conditions (data not shown). This result suggested that the therapeutic effect of anti-CD47 antibodies is unlikely to be inducing direct toxicity to the tumor cells.

### Anti-CD47 Abs inhibit spontaneous metastasis of osteosarcoma cells *in vivo*

**Figure 4:**
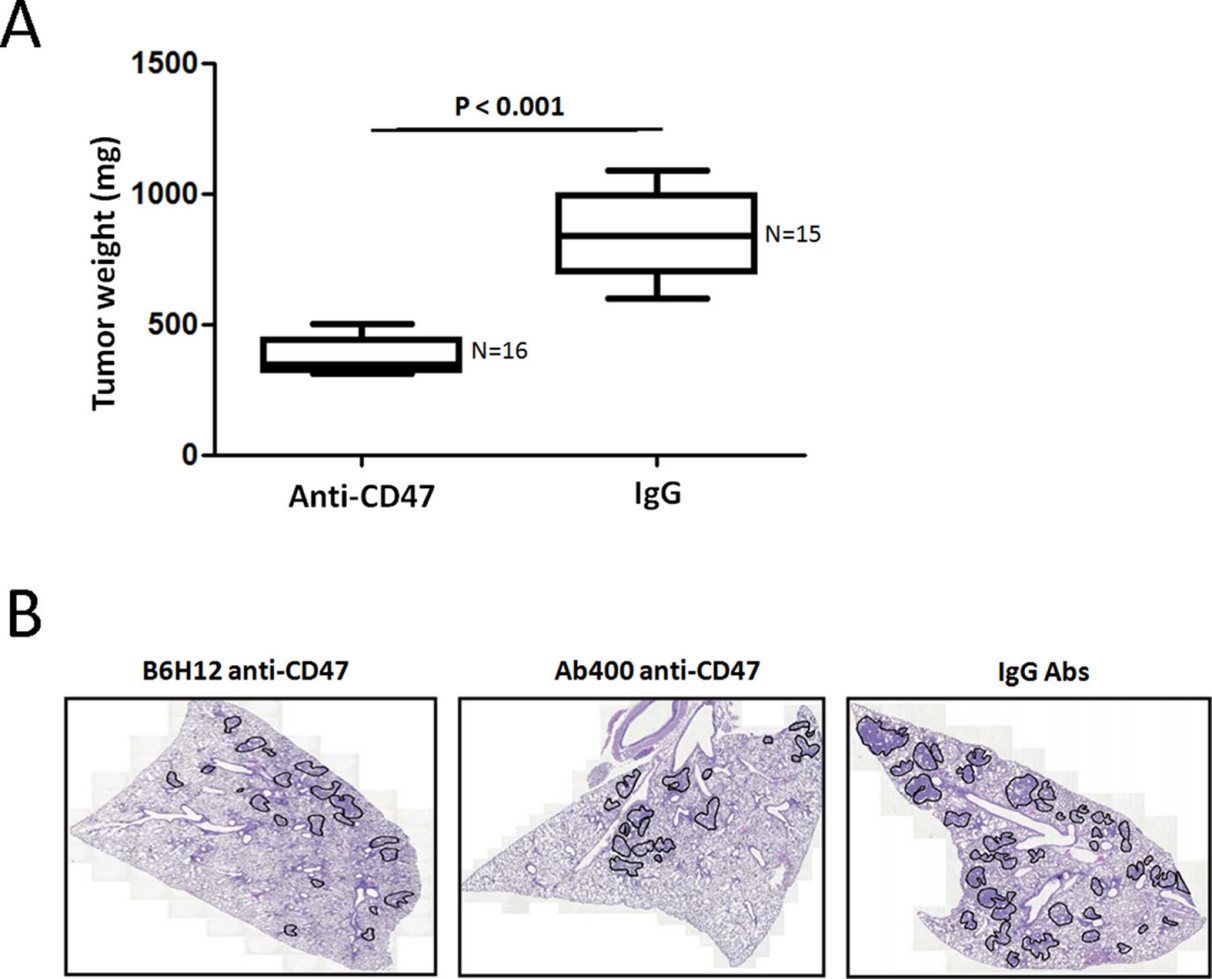
Anti-CD47 Abs inhibit spontaneous metastasis of KRIB osteosarcoma cells *in vivo* **A.** KRIB cells were injected into the tibias of nude mice. After 3 days, the mice were randomly allocated to two treatment groups (*n* = 20 each): anti-CD47 groups received i.p. injections of B6H12 Abs (100 μg) three times weekly, and the other, i.p. injections of control IgG antibody, three times weekly. After 45 days of treatment, mice were humanely killed, and the incidence of primary tumors that had developed in the tibias was determined. **B.** Tumor area/lung area was quantified within each group and at least two sections of each lung were stained with haematoxylin and eosin and analysed in a blinded manner. Tumor grading is shown in the right panel (*n* = 4 ~ 7 mice). Data in both panels were analysed by one-way ANOVA with Tukey's multiple comparison test and shown as mean ± s.e.m. Scale bar, 2 mm.

An animal model of osteosarcoma has been established using the KRIB cell line. Most of the mice with viable xenografts subsequently develop pulmonary metastasis [12]. To study the effect of anti-CD47 Abs on osteosarcoma growth *in vivo*, KRIB cells were injected into the tibias of nude mice. After 3 days, the mice were randomly allocated to two treatment groups (*n* = 20 each): anti-CD47 groups received i.p. injections of B6H12 Abs (100 μg) three times weekly, and the other, i.p. injections of control IgG antibody, three times weekly. After 45 days of treatment, mice were humanely killed, and the incidence of primary tumors that had developed in the tibias was determined. The number of mice that developed tibial tumors was similar in both treatment groups. Sixteen mice (80%) developed tumor in IgG-treated mice and 15 mice (75%) in the anti-CD47 Abs (B6H12)-treated group. All mice that had developed primary bone tumors were weighted for tumor-bearing. The mean tibial weight was lower in mice treated with anti-CD47 Abs (B6H12) (mean, 430 mg; range, 285–677 mg) than in those treated with control IgG (mean, 841 mg; range, 600–1088 mg; *P* < 0.001, Figure 4A).

The incidence of spontaneous metastasis was significantly less in anti-CD47-treated mice (11%) than it was in IgG-treated control mice (Wilcoxon's rank-sum *P* < 0.0001). In the anti-CD47-treated group, two of the 15 mice developed pulmonary metastases. However, in the IgG-treated group, 75% of the mice developed pulmonary metastases. Figure 4B illustrates the presence and absence of spontaneous lung metastasis (as determined by H&E staining) in a representative control mouse and an anti-CD47-treated mouse, respectively. Because spontaneous lung metastases occur only in mice that have established tumors from intratibially xenografted KRIB cells, the incidence of lung metastasis occurring in mice with established bone tumors was 2 of 15 (13%) in anti-CD47-treated mice and 12 of 16 (75%) in IgG-treated mice (Wilcoxon's rank-sum *P* < 0.0001 for both comparisons). These results demonstrated that anti-CD47 inhibited spontaneous pulmonary metastasis in mice bearing intratibial KRIB osteosarcoma xenografts.

### CD47 blockade using specific antibodies increases macrophage phagocytosis of osteosarcoma tumor cells

**Figure 5:**
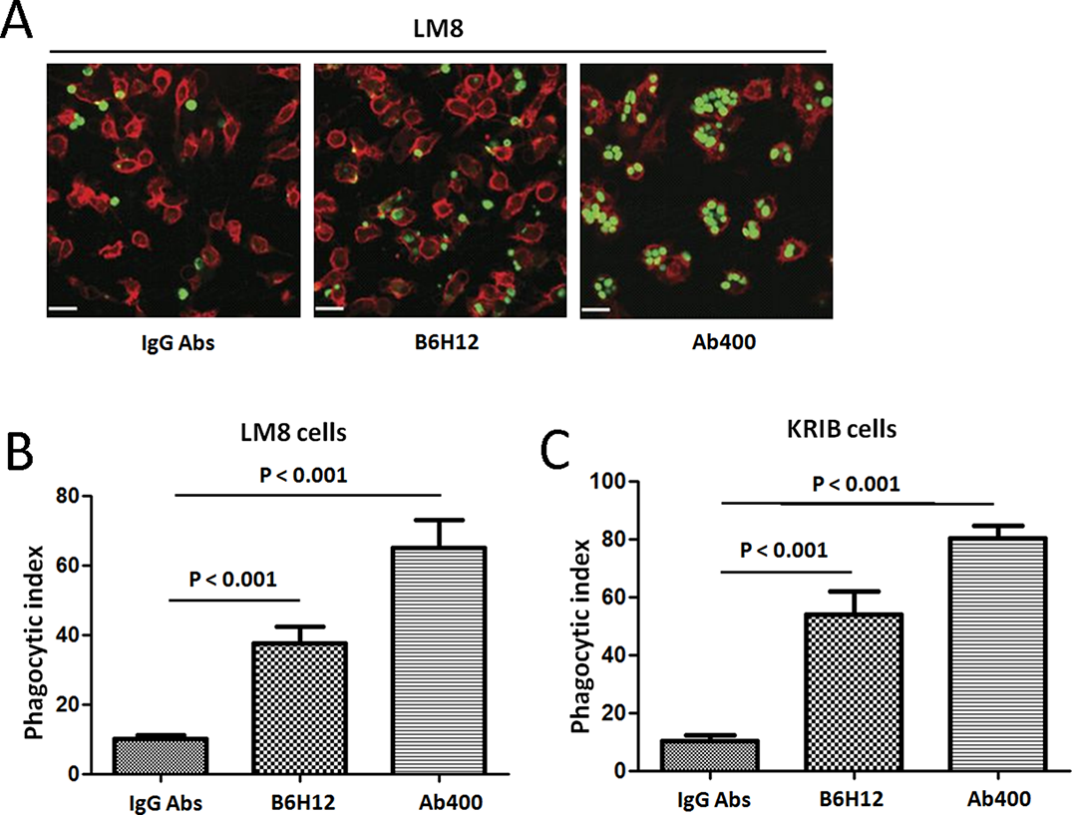
CD47 blockade using specific antibodies increases macrophage phagocytosis of osteosarcoma tumor cells **A.** LM8 tumor cells were labeled with CFSE and cultured with peritoneal-derived macrophages from NSG mice. The cells were incubated with control IgG or blocking B6H12 anti-CD47 antibody. Representative 200 × magnification images are shown for visualization by spinning disk confocal microscopy. Incubation with blocking anti-CD47 antibody resulted in increased phagocytosis of CFSE labeled LM8 cells (arrows). Bars, 25 μm. The extent of macrophage phagocytosis with each antibody was quantified for LM8 (**B.** *p* < 0.001) and KRIB cells (**C.** *p* < 0.001).

LM8 and KRIB tumor cells were labeled with carboxy fluorescein diacetate succinimidyl ester (CFSE) and co-cultured with the macrophages from NOD/SCID/IL2γ^null^ NSG mice, which harbor a SIRPα polymorphism that results in enhanced SIRPα binding to human CD47 ligand (18, 19). Elicited macrophages were obtained by injection of Brewer's thioglycollate medium into the peritoneal cavity of NSG mice. After 2 days, peritoneal fluid was extracted from these mice, and flow cytometry for macrophage markers using F4/80 and CD11b were performed. Macrophage and LM8, KRIB tumor cells incubation with control antibodies IgG resulted in low levels of tumor phagocytosis by macrophages. In contrast, CD47 blockade using B6H12 and Ab400 resulted in significantly higher rates of macrophage phagocytosis (Figure 5A–5C). These results strongly suggest that CD47 blockade *in vivo* may lead to enhanced phagocytic activity of macrophages against osteosarcoma.

## DISCUSSION

CD47 exerts its anti-phagocytic role through binding to phagocytic cells that express SIRPα [13]. Upon binding, CD47 initiates a signal transduction cascade resulting in inhibition of phagocytosis. Recently, CD47 was reported to be a marker of tumor-initiating cells in leukemia and bladder cancer [14]. In this study, we found that CD47^+^ cells have higher capacity in osteosarcoma tumorigenicity and metastasis, when compared with CD47^−^ counterparts. Similar to the studies in glioma and ovarian cancer [15], we found that a higher CD47 mRNA level in osteosarcoma samples correlated with a poorer clinical outcome. In addition, CD47 was detected in all primary and xenograft samples from osteosarcoma patients. These results have demonstrated that a CD47 population in osteosarcoma was universal. These observations suggest that CD47 may be an attractive target for potential therapeutic intervention against osteosarcoma.

Tumor-associated macrophages (TAMs) have been known for its important roles in tumor behavior, depending on their polarization [16, 17]. M1, classically activated TAMs, can mediate anticancer effects by eliciting antitumor-adaptive immunity mechanisms that include phagocytosis. In contrast, M2, alternatively activated TAMs, suppress adaptive immunity and promote a tumor microenvironment (TME) that can augment cancer progression. The TME's role as a nonneoplastic component of tumors has been studied extensively in carcinomas but remains less well characterized in sarcomas [18, 19]. In our present work, we have explored the feasibility of an alternative potential approach to treat osteosarcoma, which allows macrophages to exert their M1 phenotype by removing inhibitory factors for phagocytosis. Our results demonstrated that CD47 blockade *in vivo* may lead to enhanced phagocytic activity of macrophages against osteosarcoma (Figure 5). Future studies will address the ability of anti-CD47 Abs to eliminate established osteosarcoma metastases before and following surgical resection of the primary tumor, mimicking treatment of metastatic disease in the clinical setting.

Emerging evidence suggests TAMs support tumor progression and metastasis [20]. TAMs participate in the development of a microenvironment conducive to tumor growth through remodeling of extracellular matrix and release of factors that promote cell proliferation, angiogenesis, and migration [21]. We demonstrated that blockade of CD47 signaling also enable TAMs to attack tumor cells that they would otherwise disregard. Given that TAMs are present in large numbers within tumors, it's possible that anti-CD47 antibody therapy has the potential to restore TAM immunosurveillance and fundamentally alter the role of macrophages in tumor biology.

In conclusion, we have found that CD47 was over expressed on primary osteosarcoma. We provided evidence that antibody that block CD47 is a potential and effective treatment for osteosarcoma *in vivo*. We anticipate that CD47 therefore serves as an attractive target for tumor therapy on osteosarcoma.

## MATERIALS AND METHODS

The study has been approved by the Ethical Committee of Zhejiang Provincial People's Hospital; written informed consent was obtained from all subjects or their parents in the case of children. This work received approval from the institution ethics committee and conformed to the tenets of the Declaration of Helsinki.

### Cell lines and cell culture

SV40 immortalized human fetal osteoblastic cell line, hFOB 1.19 cells were cultured in RPMI1640 medium supplemented with 10% (v/v) FBS (Life Technologies). Murine osteosarcoma cell line, LM8 cells were maintained in DMEM (Life Technologies) supplemented with 10% fetal calf serum, and cultured at 37°C with 5% CO_2_. Human osteosarcoma cell line, KRIB cells were cultured in RPMI-1640 medium supplemented with 10% FBS. FOB 1.19 cells, LM8 cells, and KRIB cells were provided by the Cell Bank, Shanghai Institute of Biochemistry and Cell Biology, SIBS, CAS.

### Tumor xenograft models

Animal experiments were approved by the Animal Care Committee of Zhejiang Provincial People's Hospital, and were conducted according to institutional guidelines for animal care. LM8 is an isolated variant of a murine osteosarcoma cell line, Dunn osteosarcoma, which can metastasize to distant organs at a high rate [22]. For subcutaneous implantation, LM8 cells were suspended in phosphate-buffered saline (PBS), and 100 μl of cell suspension (1 × 10^6^ cells) was injected subcutaneously under the dorsal skin of C3H male mice at day 0. The C3H male mice were provided by SLRC laboratory animal company, Shanghai, China. The anti-CD47 monoclonal antibody (mAbs) B6H12 and Ab400 was purified from hybridomas. The mice were randomly divided into three groups (*n* = 20 mice/group) for B6H12, Ab400 and isotype Abs treatment. Antibodies were administrated as follow. Aliquots of 0.2 mg of B6H12, Ab400 were injected intraperitoneally into C3H mice at −7, −2, 2, 7, 14, and 19 days after LM8 transplantation. Normal rat IgG was used as a control group and injected in the same manner as for the B6H12 and Ab400 groups.

### Evaluation

Mice were sacrificed and anti-CD47 inhibitory effects were examined 4 weeks after LM8 transplantation. The wet weight of removed subcutaneous tumors was measured, and excised lungs were fixed with formalin. Fixed organs were then embedded in paraffin, sectioned (6 μm thickness), and stained with hematoxylin and eosin for histological observation. The number of metastatic colonies in lungs was counted under a light microscope (Nikon ECLIPSE 80i) with the selected midline section.

### Histological analysis

Lungs were fixed in 10% formaldehyde and sections cut at 5 μm thickness. For histological analysis of lung tumours, 2.5 μm sections from at least two different central planes of the lungs were cut, stained with haematoxylin and eosin and scanned with TissueFaxs software (TissueGnostics GmbH). Quantification of tumour area, tumour number and tumour grade was done with HistoQuest software (TissueGnostics GmbH) and visually controlled by two independent pathologists in a blind manner.

### Real-time PCR

Total RNA was isolated from tumor tissues of patients with osteosarcoma using Qiagen RNA isolation kit (Qiagen). cDNA was synthesized using SuperScript II reverse transcriptase with random hexamers (Life Technologies). To quantitate the expression of human CD47 mRNA, real-time PCR was performed using iQ SYBR Green Supermix (Bio-Rad, Hercules, CA). Melting curve analysis was done at the end of the reaction to assess the quality of the final PCR products. The threshold cycle C(t) values were calculated by fixing the basal fluorescence at 0.05 units. Three replicates were used for each sample, and the average C(t) value was calculated. The ΔC(t) values were calculated as C(t) sample - C(t) GAPDH. The N-fold increase or decrease in expression was calculated by the ΔΔCt method using the C(t)GAPDH value as the reference point.

### Western blotting

Protein samples (25 μg/lane) were subjected to SDS-PAGE and then transferred to polyvinylidene difluoride membranes (Life technologies). The resultant membranes were blocked with 5% milk-TBST for 1 h at room temperature and then incubated with anti-CD47 Ab (Abcam) overnight at 4°C, washed with TBST, incubated with appropriate secondary antibody (1:5000; Jackson ImmunoResearch) conjugated to horseradish peroxidase, washed, and visualized with ECL Western Blotting Detection Reagents (Amersham Biosciences). After stripping with Restore Western Blot Stripping Buffer (Pierce) for 20 min at room temperature, membranes were processed similarly with anti-beta-actin antibody (1:2, 000 dilution, Abcam) as a loading control.

### Immunohistochemistry

CD47 protein expression was evaluated by fluorescent immunohistochemistry on paraffin-embedded tissues. Specimens from ten patients who had undergone surgical resection for treatment of osteosarcoma were obtained under study protocol approved by the Ethical Committee of Zhejiang Provincial People's Hospital; written informed consent was obtained from all subjects. This work received approval from the institution ethics committee and conformed to the tenets of the Declaration of Helsinki. Sections (4 μm) were made from the paraffin blocks, blocked with 1% BSA and incubated with sheep anti-CD47 antibody at 0.5 μg/mL (RD Systems, AF4670) overnight at 4°C. The sections were then incubated with donkey anti-sheep IgG conjugated with FITC (RD Systems) for 1 hour, followed by nuclear counterstaining with Hoechst (Life technologies). The stained sections were imaged and immunofluorescence intensity was measured using NIS-Elements Microscope Imaging Software (Nikon).

### Flow cytometry

Cells were adjusted to a concentration of 10^6^ cells/mL in sorting buffer [1X PBS; 3% FBS (v/v); 3 mmol/L EDTA (v/v)] before analysis or sorting with a FACS Canto II or FACS Influx instrument, respectively (BD Biosciences). Cells were incubated with Abs for 30 min at 4°C in PBS containing 1% bovine serum albumin. To identify distinct cancer (stem) cells, the following antibodies were used: phycoerythrin-conjugated anti-CD4 mAb (Biomeda, Foster City, CA); CD47-APC or appropriate isotype-matched control antibodies (all from BD Biosciences). Following incubation, cells were washed, resuspended in 300 μl of PBS, and assayed using a fluorescence-activated cell sorter (FACScan; BD Bioscience). DAPI was used for exclusion of dead cells. For this experiment, at least three mice were used. Data were analyzed with FlowJo 9.2 software (Tree Star).

### Antibody preparation and MTT assay

To assess for direct potential cytotoxic effects of anti-CD47 antibody, LM8 cells were cultured, counted and placed into individual wells of a 96-well plate. IgG control, B6 h12 or Ab400 antibodies were added to the wells to final concentrations of 10 μg/mL, 3 μg/mL or 1 μg/mL. After 24 hours, 48 hours and 72 hours of incubation at 37°C, 20ug/mL MTT (Promega) was added to each well. After 4 additional hours of incubation at 37°C with MTT, the media from the wells were removed and MTT solubilization solution was added and incubated for 15 minutes, after which absorbance was measured at 590 nm with a reference filter of 620 nm.

### *In vitro* phagocytosis assay

For *in vitro* phagocytosis assay, 5 × 10^4^ macrophages were plated per well in a 24-well tissue-culture plate. Peritoneal macrophages were isolated following injection of 1 mL 3% thioglycollate medium into the peritoneal cavity of 6 week old NOD/SCID/IL2γ^null^ (NSG) mice provided by SLRC laboratory animal company, Shanghai, China. LM8 tumor cells were labeled with 2.5 μM carboxyfluorescein succinimidyl ester (CFSE) according to the manufacturer's protocol (Life Technologies). Macrophages were incubated in serum-free medium for 2 h before adding 2 × 10^5^ CFSE-labeled LM8 cells. The phagocytic index was calculated as the number of phagocytosed CFSE^+^ cells per 100 macrophages. The antibodies B6H12 and Ab400 or IgG control were added at a concentration of 10 μg/mL and incubated for 2 hours at 37°C. The macrophages were then washed and subsequently imaged using Confocal microscopy. The phagocytic index was calculated as the number of phagocytosed CFSE^+^ cells per 100 macrophages.

### Statistical analysis

All analyses were performed using the SPSS 12.0 software package (SPSS Inc). Unpaired Student's *t*-tests were used to assess statistical differences between control and B6H12-injected groups. *P*-values corresponding to < 0.05 were considered to be statistically significant.
